# Real-world systemic sequential therapy with regorafenib for recurrent hepatocellular carcinoma: analysis of 93 cases from a single center

**DOI:** 10.1186/s12876-023-02661-2

**Published:** 2023-02-01

**Authors:** Qingwei Zhu, Wei Rao, Junyu Huo, Zixiang Li, Song Wang, Wensheng Qiu, Ge Guan, Yang Xin, Ning Fan, Jinzhen Cai, Liqun Wu

**Affiliations:** 1 Liver Disease Center, The Affiliated Hospital of Qingdao University, No.59 Haier Road, Qingdao, 266003 Shandong People’s Republic of China; 2 Department of Interventional Medicine Cancer Hospital, The Affiliated Hospital of Qingdao University, Qingdao, 266003 Shandong People’s Republic of China; 3 Tumor Hospital, The Affiliated Hospital of Qingdao University, Qingdao, 266003 Shandong People’s Republic of China

**Keywords:** Hepatocellular carcinoma, Regorafenib, Combination therapy, Real-world research

## Abstract

**Background:**

Regorafenib is an oral multikinase inhibitor and became the first second-line systemic treatment for hepatocellular carcinoma (HCC) following the phase III RESORCE trial. This single-center study retrospectively analyzed the clinical data and follow-up results of patients with recurrent HCC treated with regorafenib and discussed the prognostic factors to provide guidance for clinical treatment.

**Methods:**

Ninety-three recurrent HCC patients were enrolled in the research and follow up from December 2017 to December 2020. Clinical and pathological data were collected. SPSS software v26.0 was used (Chicago, IL, USA) for statistical analysis. A two-sided *P* < 0.05 was considered statistically significant.

**Results:**

The patients included 81 males and 12 females with a median age of 57 years. Eighty-seven patients had hepatitis B virus (HBV) infection. The objective response rate (ORR) was 14.0%, and the disease control rate (DCR) was 62.4%. The median overall survival (mOS) and median time to progression (mTTP) were 15.9 and 5.0 months. Multivariate analysis showed that Child–Pugh classification, the Eastern Cooperative Oncology Group performance status (ECOG PS), the neutrophil-to-lymphocyte ratio (NLR), combined treatment, and the time from first diagnosis of HCC to second-line treatment were independent factors affecting the prognosis of recurrent HCC patients.

**Conclusions:**

This real-world study demonstrated similar findings to those of the RESORCE trial. Regorafenib could effectively improve the prognosis of patients after first-line treatment failure. Combination therapy under multidisciplinary treatment (MDT) team guidance could be effective in impeding tumor progression and improving the prognosis of recurrent HCC patients.

## Background

According to global cancer statistics from 2020, hepatocellular carcinoma (HCC) has become the sixth most common cancer and the third- leading cause of cancer-related death in the world [1]. In China, it is listed as the second-most common cancer and the fourth-most common cause of cancer-related death, with almost the same mortality and morbidity [2]. At the first diagnosis, fewer than 30% of the patients are suitable for radical treatment because of the concealed onset of HCC, and for the rest of the patients, systematic antineoplastic monotherapy or combination therapy may transform it into a resectable tumor [3]. Sorafenib, a tyrosine kinase inhibitor (TKI), has become one of the first-line treatments for HCC patients who are not suitable for local treatment [4]. However, it has been reported that 40–56% of patients receive second-line treatment due to sorafenib resistance [5]. In the RESORCE trial, a phase III trial demonstrated that regorafenib significantly improve the overall survival (OS) of HCC patients who progressed after sorafenib treatment, becoming the first TKI approved for second-line therapy [6]. Regorafenib can block the activity of protein kinases associated with angiogenesis, tumor growth, and metastasis [7, 8] and has previously been approved for the treatment of metastatic colorectal cancer and advanced gastrointestinal stromal tumors [9, 10]. Recently, in a large real-world study in South Korea, similar results were obtained [11]. In this study, we attempted retrospectively analyzed clinical data and follow-up results of patients with recurrent HCC treated with regorafenib to identify the factors affecting the prognosis of these patients.

## Methods

### Enrolled cases

A total of 106 patients with recurrent HCC treated with regorafenib were reviewed from December 2017 to December 2020. A total of 93 met the inclusion criteria, including 81 males and 12 females with an average age of 57.3 years (31–80 years). All the patients were cared for in the outpatient department of the Affiliated Hospital of Qingdao University, and the relevant clinical data and follow-up results were obtained from the His system of our hospital and telephone calls. This study was conducted in accordance with the ethical guidelines of the Declaration Helsinki and was approved by the Medical Ethics Committee of the Affiliated Hospital of Qingdao University (QYFYKYLL2018-12). The requirement for informed consent was waived.

The inclusion criteria were as follows: (1) initial treatment was liver transplantation, hepatectomy or ablation; (2) Child–Pugh class A or B; (3) Eastern Cooperative Oncology Group performance status (ECOG PS) score ≤ 2; and (4) exposure time of regorafenib ≥ 56 days.

The exclusion criteria were as follows: (1) nonradical treatment at the first diagnosis; (2) non-HCC at the first diagnosis and recurrence; (3) ECOG PS > 2; and (4) Child–Pugh C.

### Targeted drug management and efficacy evaluation

A standard dose of regorafenib of 80–160 mg (two-four tablets of 40 mg each) was administered orally for the first three weeks, followed by a holiday week for each 4-week cycle. Dose modification and interruption were performed at the discretion of the attending physician(s) based on the type and severity of the adverse events. A dose reduction of up to 80 mg once daily was allowed as per the protocol of the RESORCE trial. Treatment was followed until disease progression, death, or intolerable toxicity [6].

Efficacy evaluation: The efficacy was evaluated 8 weeks after treatment and was classified as complete response (CR), partial response (PR), stable disease (SD) or progressive disease (PD). Response was assessed based on the modified Response Evaluation Criteria in Solid Tumors 1.1 (mRECIST 1.1)-that is, the presence of new tumor lesions or vascular invasion inside or outside the liver or the enlargement of the original tumor lesions ≥ 20% compared with the baseline [12, 13].

### Follow-up

Ten days posttreatment, the patients were followed up, and the general situation and adverse events were recorded, mainly by telephone follow-up. Liver function examination and urine analysis were performed on the 4th week and dynamic enhanced computed tomography (CT) or dynamic enhanced magnetic resonance imaging (MRI) were performed on the 8th week, and followed up every 2 months. The progression of HCC was diagnosed by enhanced CT, enhanced MRI or positron emission tomography-CT (PET-CT) according to the mRECIST1.1 standard. OS was defined as the time from the initiation of treatment with regorafenib to death from any cause, and the time to progression (TTP) was defined as the time from the initiation of treatment with regorafenib to the date of disease progression. The follow-up period of this group of patients ended on December 31, 2021, or the time of death.

### Statistical analysis

SPSS software v26.0 was used (Chicago, IL, USA) for statistical analysis. The quantitative data were analyzed by t-test, the Kaplan–Meier method for survival analysis, the chi-square test and log-rank test for intergroup comparison, and univariate and multivariate Cox proportional hazard models were used to identify the factors associated with survival. A *P* value of < 0.05 was considered statistically significant.

## Results

### Baseline characteristics

The baseline characteristics of all enrolled patients (n = 93), including 81 males and 12 females, are shown in Table 1. Eighty-seven (93.5%) patients were complicated with hepatitis B virus (HBV) infection. All except 8 patients (Child–Pugh B) were classified as Child–Pugh A at the start of regorafenib administration. Most of the patients were classified as having an ECOG PS score of 0 or 1 (73/93, 78.5%). After the first diagnosis of HCC, 17 patients underwent liver transplantation (LT), 63 patients underwent surgical resection, and 13 patients underwent radiofrequency ablation (RFA). Eighty-six patients (92.5%) were treated with sorafenib, and the rest were treated with lenvatinib. During treatment with regorafenib, 60 patients received at least one other treatment, including reresection, RFA, transarterial chemoembolization (TACE), and immune checkpoint inhibitors (ICIs) (Table 1).

**Table 1:** Baseline characteristics and survival analysis of HCC patients treated with regorafenib

At the start of regorafenib	n/n	Kaplan–Meier		Multivariate Cox	
		mOS	*P*	RR (95%CI)	*P*
Gender (Male/Female)	81/12	15.9/12.2	0.686		
Age (≤ 60/ > 60 years)	60/33	18.3/15.4	0.495		
Alcoholism (No/Yes)	59/34	15.7/15.9	0.945		
Hypertension (No/Yes)	72/21	16.9/12.2	0.877		
Diabetes (No/Yes)	80/13	15.9/15.4	0.995		
Portal hypertension(No/Yes)	64/29	15.7/16.9	0.603		
HBV infection (No/Yes)	6/87	8.0/16.9	0.077		
Child–Pugh (A/B)	85/8	16.9/7.3	0.032	2.802 (1.136–6.914)	0.025
Blood lymphocyte (≥ 1.0/ < 1.0 × 10^9^/L)	52/41	19.8/14.2	0.130		
NLR (< 2.5/ ≥ 2.5)	37/56	22.8/12.7	0.002	1.981 (1.105–3.551)	0.022
ALB (< 35/ ≥ 35 g/L)	12/81	11.6/15.9	0.098		
TBil (< 17.1/ ≥ 17.1 µmol/L)	50/43	15.1/16.9	0.498		
GGT (≤ 60/ > 60U/L)	53/40	21.2/14.1	0.070		
ECOG PS (≤ 1/2)	73/20	20.5/8.0	< 0.001	3.016 (1.664–5.469)	< 0.001
AFP (< 400/ ≥ 400 ng/mL)	62/31	15.9/15.1	0.916		
BCLC stage (B/C)	29/64	22.1/15.2	0.105		
Vascular invasion by naked eye (No/Yes)	86/7	16.9/12.2	0.510		
Extrahepatic metastasis (No/Yes)	30/63	16.8/15.2	0.064		
Pre-treatment (within 2 months, Yes/No)	48/45	19.7/15.2	0.744		
Adverse events (0–1/ ≥ 2 grade)	49/44	20.5/14.5	0.220		
Proteinuria (No/Yes)	64/29	15.9/15.4	0.893		
Combination therapy (No/Yes)	33/60	11.6/19.8	0.012	0.522 (0.305–0.891)	0.017
*At the first diagnosis of HCC*					
BCLC stage (A/B-C)	43/50	18.3/15.1	0.648		
Vascular invasion (No/Yes)	79/14	18.3/8.7	0.068		
Microvascular invasion (No/Yes)	28/47/18	22.1/14.2	0.060		
Treatment (LT/hepatectomy/RFA)	17/63/13	22.8/15.9/15.4	0.898		
Time from first diagnosis to regorafenib (> 24 months/ ≤ 24 months)	46/47	14.2/22.1	0.024	0.463 (0.260–0.824)	0.009
First-line systemic therapy (Sorafenib/others)	86/7	16.9/15.7	0.387		
Time from first line to regorafenib (≤ 6.0 months/ > 6.0 months)	36/57	19.8/14.5	0.319		

*HBV* hepatitis B virus, *NLR* neutrophil-to-lymphocyte ratio, *ALB* albumin, *TBil* total bilirubin, *GGT* γ-Glutamyl transferase, *ECOG PS* Eastern Cooperative Oncology Group performance status, *AFP* Alpha-fetoprotein, *BCLC* Barcelona Clinic Liver Cancer

### Effectiveness and safety

**Fig. 1:**
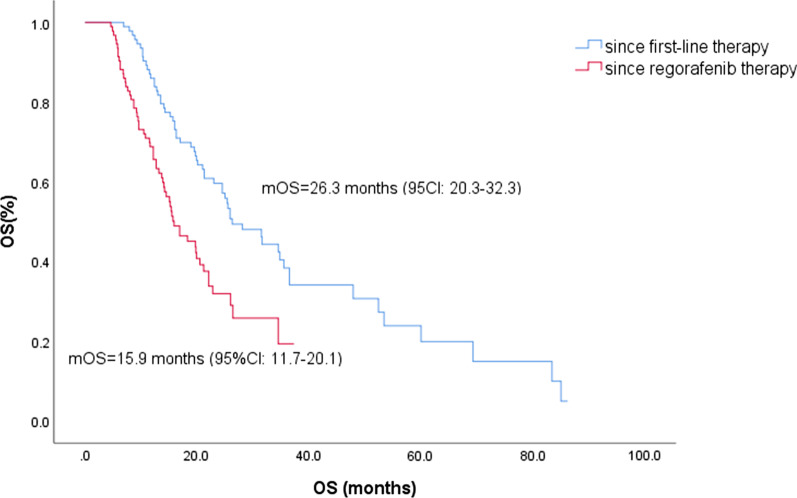
OS in patients since regorafenib and first-line systemic therapy

With regorafenib, the median follow-up time of 93 patients with HCC was 14.5 months (*95%CI* 13.0–16.9 months), the median OS (mOS) was 15.9 months (*95%CI* 11.7–20.1 months), and the median TTP (mTTP) was 5.0 months (*95%CI* 4.1–5.9 months). The survival rates at 6.0, 12.0, 18.0 and 24.0 months were 91.4, 68.8, 46.4 and 31.9%, respectively. The median duration of regorafenib administration was 9.2 months (*95%CI* 5.9–12.0 months). In the exploratory analysis after first-line systemic treatment, the mOS was 26.3 months (*95%CI* 20.3–32.3 months), and the 1-year, 2-year, 3-year and 5-year OS rates were 86.0, 59.6, 38.4 and 19.9%, respectively (Fig. 1, Table 2). By the end of follow-up, a total of 35 patients were found to have PD 8 weeks after taking regorafenib, and the disease control rate (DCR) was 62.4% (Table 2). At least one adverse event occurred in 87 patients who received regorafenib, including hand-foot skin reaction (n = 22, 23.7%), weakness (n = 17, 18.3%), skin rash (n = 16, 17.2%), diarrhea (n = 7, 7.5%) and hypertension (n = 7, 7.5%). Ten patients had grade 3–4 adverse events, including weakness (n = 1, 1.1%) and skin rash (n = 2, 2.2%). Three patients withdrew from regorafenib due to uncontrollable headache, hepatocyte dysfunction and myelosuppression. The adverse reactions of other patients were controlled by life and drug intervention and dose reduction (Table 3).

**Table 2:** Efficacy data on regorafenib therapy

Variable	Total (n = 93)
*Response by mRECIST, n (%)*	
Complete response	2 (2.2)
Partial response	11 (11.8)
Stable disease	45 (48.4)
Progressive disease	35 (37.6)
Objective response rate, n (%)	13 (14.0)
Disease control rate, n (%)	58 (62.4%)
Time of first-line drug treatment, median months (*95%CI*)	6.9 (6.0–8.8)
Time of regorafenib treatment, median months (*95%CI*)	9.2 (5.9–12.0)
Time of follow up, median months (*95%CI*)	14.5 (13.0–16.9)
Overall survival, median months (*95%CI*)	15.9 (11.7/20.1)
Overall survival from first-line drug treatment, median months (*95%CI*)	26.3 (20.3/32.3)
Time to progression, median months (*95%CI*)	5.0 (4.1/5.9)

*mRECIST* modified Response Evaluation Criteria in Solid Tumors, *CI* Confidence Interval

**Table 3:** Adverse events following regorafenib therapy

	Any grade, Number (%)	Grade ≥ 3, Number (%)
Hand-foot skin reaction	22 (23.7%)	0
Weakness	17 (18.3%)	1 (1.1%)
Skin rash	16 (17.2%)	2 (2.2%)
Diarrhea	7 (7.5%)	0
Hypertension	7 (7.5%)	0
Mucositis	5 (5.3%)	0
Anorexia	3 (3.2%)	1 (1.1%)
Proteinuria	3 (3.2%)	0
Renal dysfunction	3 (3.2%)	2 (2.2%)
Hepatocyte dysfunction	2 (2.2%)	2 (2.2%)
Headache	1 (1.1%)	1 (1.1%)
Myelosuppression	1 (1.1%)	1 (1.1%)

Multivariate analysis showed that Child–Pugh classification, ECOG PS, neutrophil-to-lymphocyte ratio (NLR), use of combined therapy, and time from the first diagnosis of HCC to treatment with regorafenib were predictive factors for OS (*P* < 0.05) (Table 1).

### Subgroup analysis

**Fig. 2:**
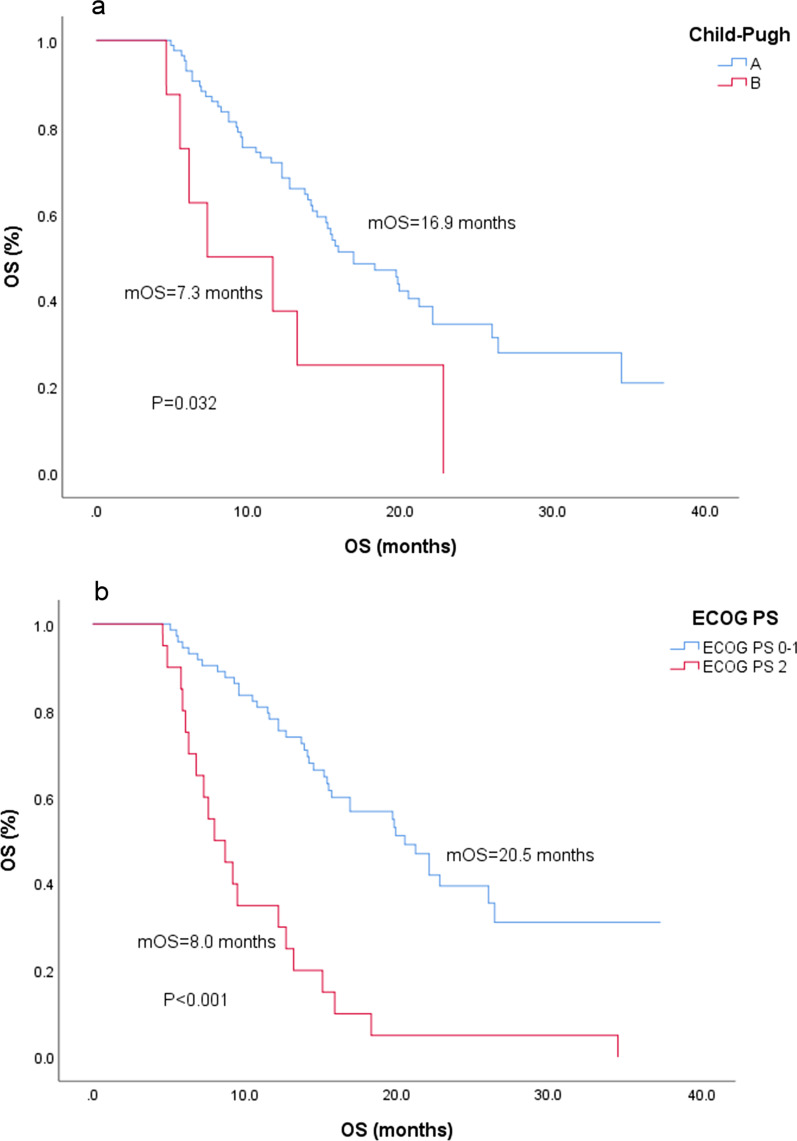

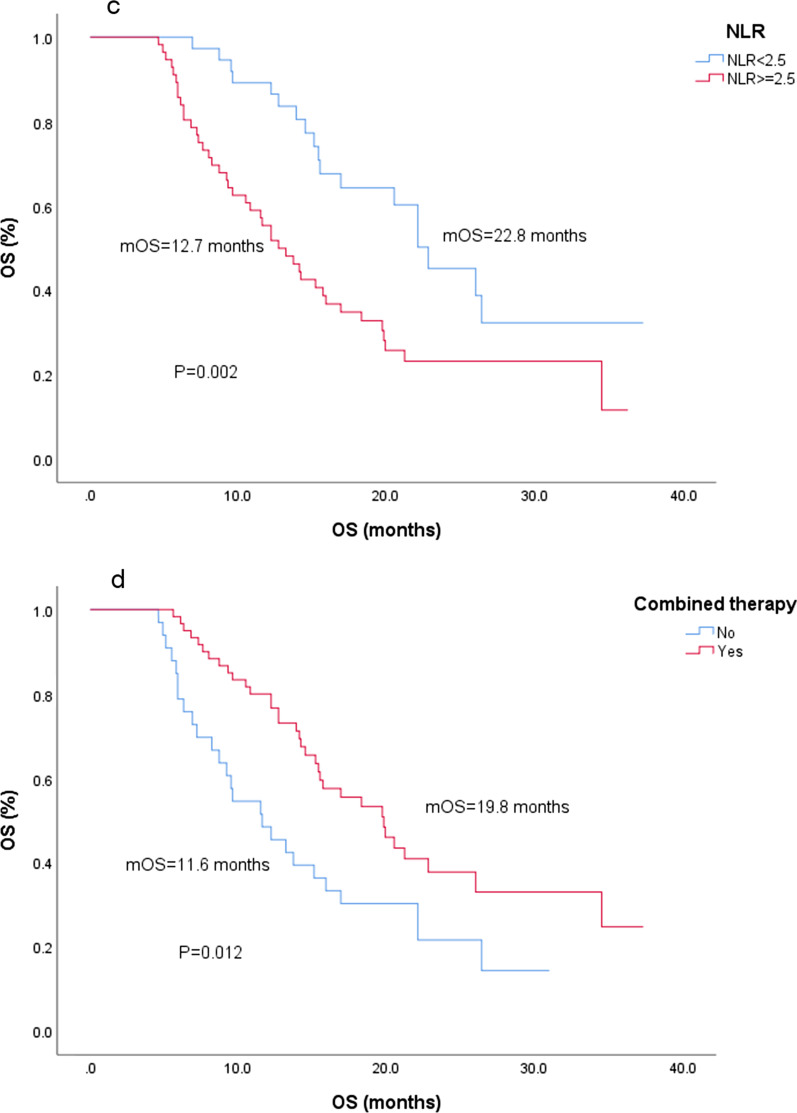

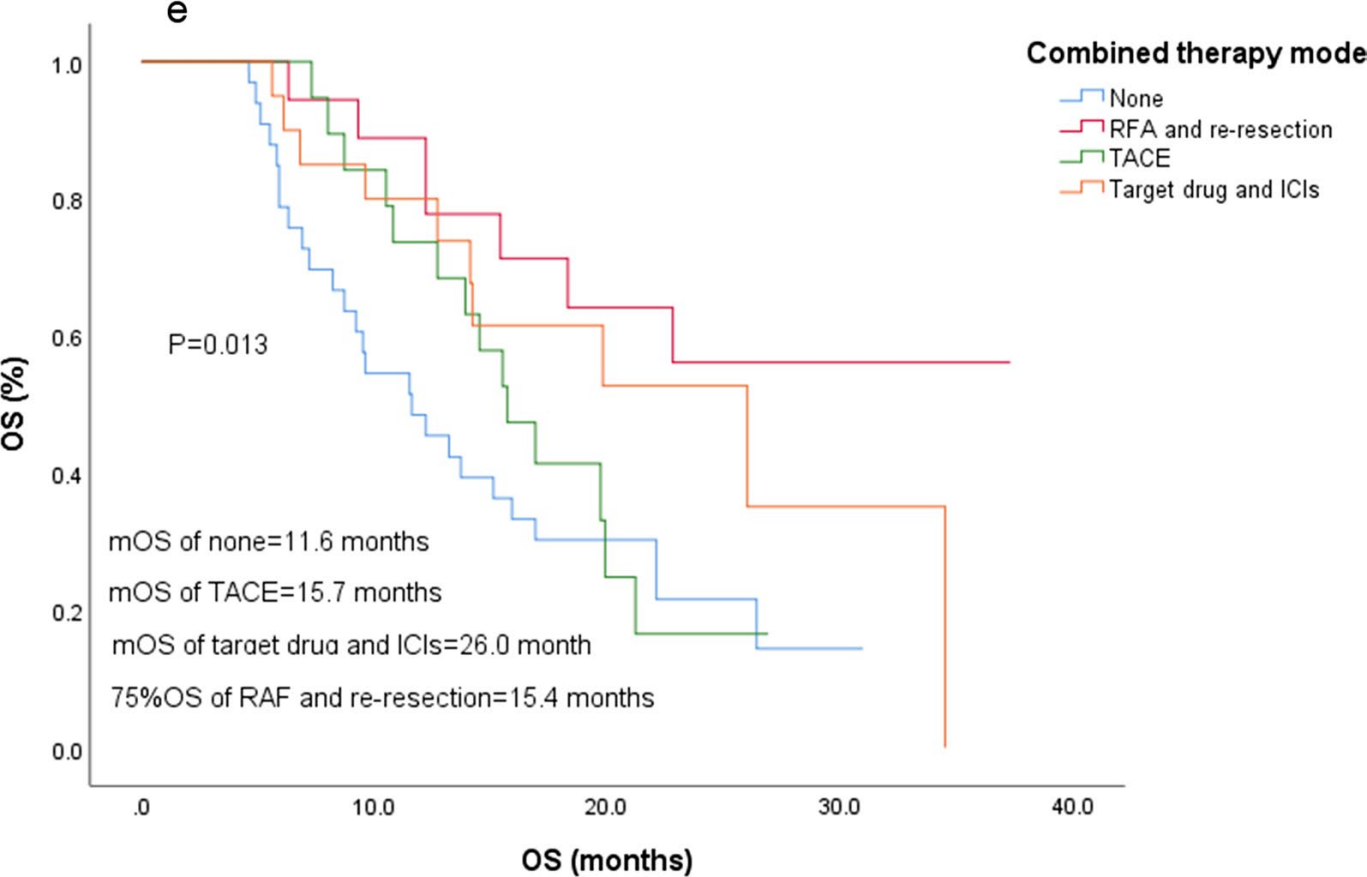
Time from regorafenib administration of factors (**a**) Child–Pugh classification, (**b**) ECOG PS, (**c**) NLR, (**d**) combined therapy, and (**e**) combined therapy mode

Eighty-five patients were classified as Child–Pugh A, and 8 patients were classified as Child–Pugh B; the 1-year survival rates for these groups were 71.8% and 37.5%, respectively, with a significant difference (*P* = 0.032) (Fig. 2a). In addition, there was no correlation between Child–Pugh classification and BCLC stage (*P* = 1.000), GGT (*P* = 0.965), AFP (*P* = 0.513), extrahepatic metastasis (*P* = 0.949) or vascular invasion (*P* = 1.000).

The 1- year survival rates of patients with ECOG PS ≤ 1 (73 patients) and ECOG PS = 2 (20 patients) were 78.1 and 35.0%, respectively, and 2-year survival rate for these groups were 39.5 and 5.0%, respectively (*P* < 0.001) (Fig. 2b). The incidence of grade 2 or higher adverse events in patients with ECOG PS 2 (15/20, 75.0%) was higher than that in patients with ECOG PS 0–1 (29/73, 39.7%) (χ^2^ = 7.836, *P* = 0.005). ECOG PS was not correlated with serum albumin (*P* = 0.489), transpeptidase levels (*P* = 0.839), portal hypertension (*P* = 0.897), BCLC stage (*P* = 0.897) or Child–Pugh classification (*P* = 0.109).

We found that there was a significant difference in OS between patients with NLR ≥ 2.5 and NLR < 2.5, and the 1- and 2-year survival rates were 55.4% and 23.2% and 89.2% and 45.2% respectively (*P* = 0.002) (Fig. 2c). The NLR was not related to other factors (*P* > 0.005).

Eighty-five patients (91.4%) presented tumor progression during regorafenib administration, of whom 60 patients (64.5%) received combined therapy, and the 1-year and 2-year survival rates were 80.0 and 37.8%, respectively, which were significantly higher than those of patients who did not receive combined therapy (48.5 and 21.6%, *P* = 0.012). The mOS times were 19.8 and 11.6 months (Fig. 2d). Combination therapy included RFA or reresection in 18 patients, only TACE in 19 patients, combined with other TKIs or ICIs in 22 patients and radiotherapy in 1 patient; the 1-year survival rates were 88.9, 73.7, 77.3 and 100%, respectively. More than half of the RFA or reresection group survived until the end of the study, with 15.4 months of 75% OS and 27.3 months as the average OS (Fig. 2e). Among the 22 patients treated with TKIs and/or ICIs, 21 received ICIs. The mOS was 26.0 months, with 10 patients surviving at the end of follow-up.

Seventeen patients (18.3%) received regorafenib treatment after LT, and 10 died during the follow-up period. The mOS times of these patients and those without LT were 22.8 months and 15.9 months, respectively, and there was no significant difference between them (*P* = 0.731). We noted that the BCLC stage C of the 17 patients was significantly higher than that of non-LT patients (94.1 and 63.2%, *P* = 0.013). Eleven patients were treated with combined therapy, including RFA in 5, reresection in 2, TACE in 2 and ICIs in 2, and 6 patients were treated with noncombined therapy.The 1-year survival rates were 72.7% and 33.3%, respectively, which were not statistically significant (*P* = 0.085), but a greater sample size and a longer follow-up period are required for reassessment.

## Discussion

In this study, we reviewed patients with recurrent HCC in our hospital and evaluated the efficacy of regorafenib and prognostic factors after first-line systemic therapy failure. Unlike other study samples, these patients had undergone surgery and first-line drug treatment; they may have better liver function, PS scores and other indicators than other patients, and they may prefer more aggressive treatment strategies. Therefore, this study may record better results. The mOS was 15.9 months (*95%CI* 11.7–20.1), the mTTP was 5.0 months (*95%CI* 4.1–5.9), and the mOS since first-line systemic therapy was 26.3 months (*95%CI* 20.3–32.3), which were all slightly better than 11.08 months, 3.2 months, and 26.0 months found in previous research [14, 15]. These findings indicate the potential effectiveness of combined therapy. Eighty-seven patients (93.5%) had at least one adverse event, of which 10 (10.8%) had grade 3–4 adverse events, and the type and incidence were similar to those in Lee's study (15.8%) [11]. The ORR and DCR of the patients enrolled in the group were 14.0 and 62.4%, respectively, which were similar to 11 and 65% of the RESORCE trial. Overall, this study indicated that regorafenib showed tolerable safety and valuable effectiveness.

At present, Child–Pugh classification and ECOG PS have been recognized as important factors affecting the prognosis of patients [16, 17]. In this study, we found that the patients with high ECOG PS scores had more adverse events (χ^2^ = 7.836, *P* = 0.005) and earlier drug withdrawal (χ^2^ = 17.201, *P* < 0.001). In addition, the number of patients with BCLC stage C after LT was significantly higher than that of non-LT patients (94.1 and 63.2%, *P* = 0.013), but there was no difference in OS (*P* = 0.731) or adverse events (*P* = 0.272). Combined with the findings of Iavarone et al. [18], we believe that regorafenib is safe and effective in patients with recurrent HCC after LT.

Inflammatory cell infiltration is related to tumorigenesis and development [19]. Lymphocytes are involved in inhibiting the proliferation and migration of tumor cells and inducing cytotoxic cell death. In contrast, neutrophils determine the development and invasiveness of tumors [20]. Therefore, a higher NLR may reflect a poorer prognosis. However, no optimal cutoff value has been reported for the NLR. Bruix et al. [16] used the median NLR as the dividing line in a study of prognostic factors of sorafenib, and a meta-analysis of more than 3000 patients with HCC [21] revealed that the threshold NLR value ranged from 1.9 to 5, and NLR > 3 was a better predictor of OS than NLR between 2 and 2.9. In this study, we found that this value is an important predictor of OS (22.8 months/12.7 months, *P* = 0.002) when taking NLR = 2.5 as the cutoff value, which may be due to the dissimilar sample of patients assessed.

The purpose of combined therapy is to resect the tumor and reduce the tumor burden. Sixty patients (64.5%) received combined therapy under multidisciplinary treatment (MDT), of which 15 patients received a combination of more than two regimens, and the incidence of adverse events did not increase with the increase in treatment (*P* = 0.766). HCC has a high degree of heterogeneity, and increasing treatment models and timely multidisciplinary discussions will help to improve the treatment accuracy and compliance of each patient.

A recent study showed [22] that regorafenib could enhance antitumor immunity by reversing the M2 polarization of tumor-associated macrophages, which provides a theoretical basis for the combination of regorafenib and ICIs in the treatment of HCC. The mOS of 26 patients who received combined treatment with ICIs (including multiple combinations) was significantly longer than that of those without combined therapy (20.5 months/11.6 months, *P* = 0.020). Three patients achieved PR, and the DCR was 61.5%. It was suggested that there was a potential synergistic effect between ICIs and standard regimens with immunomodulatory effects. However, the TTP and OS of the other 19 patients who received TACE treatment did not show significant advantages (*P* = 0.324, *P* = 0.431). In the recent TACTICS study [23], the newly established specific end point of TACE was used to define the "time to untreatable progression (TTUP)", that is, "no TACE progression or TACE failure". Finally, it was found that the median PFS of the “TACE + sorafenib” group was significantly longer than that of the simple TACE group (25.2 months vs. 13.5 months, *P* = 0.006), and the median TTUP was also significantly prolonged (26.7 months vs. 20.6 months, *P* = 0.02) with controllable toxicity.

Conversed therapy is an important way to improve the survival of patients with advanced HCC [24]. A total of 107 patients with advanced HCC treated with lenvatinib were followed up, of which 16 (15.0%) underwent surgery and 9 (9.4%) received R0 resection; these patients had a longer OS (*P* = 0.002) [25]. In addition, there are a number of case reports describing that patients benefit significantly from TKI drugs for the conversion of advanced HCC [26–29]. In this study, one patient received combination treatment with regorafenib and ICIs; this patient was found to have abdominal metastasis 3 months after HCC resection, and then, the abdominal tumor was resected. The OS was 26.3 months. Another patient was also found to have abdominal and colon metastasis. After the administration of sorafenib, the metastatic focus was resected and then treated with regorafenib combined with ICI adjuvant therapy, and a 19.8-month OS was obtained. By the end of follow-up, the 2 patients were found to have achieved PR and CR. In this study, 18 patients (19.1%) achieved downstaging of tumors, 6 patients underwent reresection after regorafenib (5 in R0) and 12 in RFA, and more than half of them survived until the end of follow-up. The 75% OS was 15.4 months, and the average OS reached 27.3 months.

In the subgroup analysis of the study, the prognosis of patients with recurrent HCC treated with conversion therapy seemed to be better than that of patients treated with ICIs or TACE. Systematic treatment degrades the tumor stage of some patients with strict screening, which represents a bridge to successful surgery or RFA therapy. Some patients could still be evaluated for PR or even CR after two or even multiple operations, which provided us with the hope of cure for patients with recurrent HCC. However, due to the unique biological behavior of HCC and the low sensitivity to TKIs and ICIs, the surgical conversion rate of advanced HCC is not ideal, and how to choose suitable patients and the opportunity for operation need to be studied in follow-up work.

When we observed the patient's disease progression, we did not immediately withdraw regorafenib, but combined with other treatments. However, combination therapy will dilute the efficacy of regorafenib, and as a retrospective study, we cannot determine how much of it accounts for. In the real world, combination therapy strategies are very common, but the timing, methods and side effects of combination therapy are waiting to be studied. Therefore, one of the conclusions of our study is that the combination therapy discussed by MDT, including regorafenib, can be more effective than regorafenib alone, and the side effects can be controlled.

Different from the RESORCE test, we take exposure time of regorafenib ≥ 56 days as one of the inclusion criteria, which may cause selection bias. However, during the research, we found that the first imaging reexamination of the patients was 8 weeks after treatment with regorafenib, which was an important basis for us to evaluate the curative effect; we also found that this standard can effectively exclude patients with poor compliance and premature death due to rapid tumor progression. In a sense, the standard can reject certain bias.

Nevertheless, there are several limitations in this research. First, this study was based on a retrospective analysis of limited data from a single institution. The size of the participants was small, and selection bias and a lack of data were inevitable. The conclusions need to be verified in a multicenter large-sample prospective study. Second, almost all the patients were infected with HBV, which was consistent with the characteristics of the Chinese HCC population, but for HCC patients with HCV infection, alcoholic hepatitis, nonalcoholic hepatitis and other causes, more follow-up data are needed to confirm our conclusions. Third, the treatment plan for postoperative recurrence in this study was based on the latest clinical guidelines at that time, combined with the tumor characteristics and clinical information of the patients, but in the end, it was limited by the patients' preference for less invasive procedures and economic conditions, and there may be bias. The above factors may reduce the quality of the conclusions obtained in this study. In the next step, a larger, multicenter real-world study with longer follow-up is needed to improve the level of evidence of the conclusions of this study, to provide a basis for the formulation of individual and accurate treatment plans and to improve the prognosis of patients with HCC.

## Conclusions

This study recruited a group of patients who received combined treatment based on regorafenib after curative therapies, failed, received first-line systematic therapy and failed again. For them regorafenib has acceptable efficacy and tolerance. Importantly, we discussed the advantages of combination therapy. In the whole course of regorafenib administration, a timely and effective MDT can optimize the treatment plan and prolong the OS of patients, while an effective systemic treatment program followed by surgery can even result in some patients with recurrent HCC achieving CR.

## Data Availability

The datasets generated during and analyzed during the current study are obtained from the His system of the Affiliated Hospital of Qingdao University. But they are related to the privacy of patients, so they will not be shared.

## Abbreviations

HCC: Hepatocellular carcinoma
HBV: Hepatitis B virus
ORR: Objective response rate
DCR: Disease control rate
mOS: Median overall survival
mTTP: Median time to progression
ECOG PS: Eastern Cooperative Oncology Group performance status
NLR: Neutrophil-to-lymphocyte ratio
MDT: Mutidisciplinary treatment
TKI: Tyrosine kinase inhibitor
CR: Complete response
PR: Partial response
SD: Stable disease
PD: Progressive disease
LT: Liver transplantation
RFA: Radiofrequency ablation
TACE: Transarterial chemoembolization
ICIs: Immune checkpoint inhibitors
ALB: Albumin
TBil: Total bilirubin
GGT: γ-Glutamyl transferase
AFP: Alpha-fetoprotein
BCLC: Barcelona Clinic Liver Cancer

## References

[1] Sung H, Ferlay J, Siegel RL, Laversanne M, Soerjomataram I, Jemal A (2021). Global Cancer Statistics 2020: GLOBOCAN estimates of incidence and mortality Worldwide for 36 Cancers in 185 Countries. CA Cancer J Clin.

[2] Cao W, Chen HD, Yu YW, Li N, Chen WQ (2021). Changing profiles of cancer burden worldwide and in China: a secondary analysis of the global cancer statistics 2020. Chin Med J (Engl).

[3] Zhu XD, Huang C, Shen YH, Ji Y, Ge NL, Qu XD (2021). Downstaging and Resection of Initially Unresectable Hepatocellular Carcinoma with Tyrosine Kinase Inhibitor and Anti-PD-1 Antibody Combinations. Liver Cancer.

[4] Cheng AL, Kang YK, Chen Z, Tsao CJ, Qin S, Kim JS (2009). Efficacy and safety of sorafenib in patients in the Asia-Pacific region with advanced hepatocellular carcinoma: a phase III randomised, double-blind, placebo-controlled trial. Lancet Oncol.

[5] Trevisani F, Brandi G, Garuti F, Barbera MA, Tortora R, Casadei Gardini A (2018). Metronomic capecitabine as second-line treatment for hepatocellular carcinoma after sorafenib discontinuation. J Cancer Res Clin Oncol.

[6] Bruix J, Qin S, Merle P, Granito A, Huang Y-H, Bodoky G (2017). Regorafenib for patients with hepatocellular carcinoma who progressed on sorafenib treatment (RESORCE): a randomised, double-blind, placebo-controlled, phase 3 trial. The Lancet.

[7] Wilhelm SM, Dumas J, Adnane L, Lynch M, Carter CA, Schutz G (2011). Regorafenib (BAY 73–4506): a new oral multikinase inhibitor of angiogenic, stromal and oncogenic receptor tyrosine kinases with potent preclinical antitumor activity. Int J Cancer.

[8] Abou-Elkacem L, Arns S, Brix G, Gremse F, Zopf D, Kiessling F (2013). Regorafenib inhibits growth, angiogenesis, and metastasis in a highly aggressive, orthotopic colon cancer model. Mol Cancer Ther.

[9] Demetri GD, Reichardt P, Kang YK, Blay JY, Rutkowski P, Gelderblom H (2013). Efficacy and safety of regorafenib for advanced gastrointestinal stromal tumours after failure of imatinib and sunitinib (GRID): an international, multicentre, randomised, placebo-controlled, phase 3 trial. Lancet (London, England).

[10] Grothey A, Van Cutsem E, Sobrero A, Siena S, Falcone A, Ychou M (2013). Regorafenib monotherapy for previously treated metastatic colorectal cancer (CORRECT): an international, multicentre, randomised, placebo-controlled, phase 3 trial. Lancet (London, England).

[11] Lee MJ, Chang SW, Kim JH, Lee YS, Cho SB, Seo YS (2021). Real-world systemic sequential therapy with sorafenib and regorafenib for advanced hepatocellular carcinoma: a multicenter retrospective study in Korea. Invest New Drugs.

[12] Reig M, Rimola J, Torres F, Darnell A, Rodriguez-Lope C, Forner A (2013). Postprogression survival of patients with advanced hepatocellular carcinoma: rationale for second-line trial design. Hepatology.

[13] Ogasawara S, Chiba T, Ooka Y, Suzuki E, Kanogawa N, Saito T (2016). Post-progression survival in patients with advanced hepatocellular carcinoma resistant to sorafenib. Invest New Drugs.

[14] Facciorusso A, Abd El Aziz MA, Sacco R (2019). Efficacy of regorafenib in hepatocellular carcinoma patients: a systematic review and meta-analysis. Cancers (Basel).

[15] Finn RS, Merle P, Granito A, Huang YH, Bodoky G, Pracht M (2018). Outcomes of sequential treatment with sorafenib followed by regorafenib for HCC: additional analyses from the phase III RESORCE trial. J Hepatol.

[16] Bruix J, Cheng AL, Meinhardt G, Nakajima K, De Sanctis Y, Llovet J (2017). Prognostic factors and predictors of sorafenib benefit in patients with hepatocellular carcinoma: analysis of two phase III studies. J Hepatol.

[17] Fung AS, Tam VC, Meyers DE, Sim HW, Knox JJ, Zaborska V (2020). Second-line treatment of hepatocellular carcinoma after sorafenib: characterizing treatments used over the past 10 years and real-world eligibility for cabozantinib, regorafenib, and ramucirumab. Cancer Med.

[18] Iavarone M, Invernizzi F, Czauderna C, Sanduzzi-Zamparelli M, Bhoori S, Amaddeo G (2019). Preliminary experience on safety of regorafenib after sorafenib failure in recurrent hepatocellular carcinoma after liver transplantation. Am J Transpl.

[19] Zhou C, Wu Y, Jiang L, Li Z, Diao P, Wang D (2018). Density and location of CD3(+) and CD8(+) tumor-infiltrating lymphocytes correlate with prognosis of oral squamous cell carcinoma. J Oral Pathol Med.

[20] Bang Y, Yoo C, Lonardi S, Kim HD, Vivaldi C, Rimini M (2021). Sequential treatment of Sorafenib-Regorafenib versus sorafenib-physician's choice: a propensity score-matched analysis. Target Oncol.

[21] Xiao WK, Chen D, Li SQ, Fu SJ, Peng BG, Liang LJ (2014). Prognostic significance of neutrophil-lymphocyte ratio in hepatocellular carcinoma: a meta-analysis. BMC Cancer.

[22] Ou DL, Chen CW, Hsu CL, Chung CH, Feng ZR, Lee BS (2021). Regorafenib enhances antitumor immunity via inhibition of p38 kinase/Creb1/Klf4 axis in tumor-associated macrophages. J Immunother Cancer.

[23] Kudo M, Ueshima K, Ikeda M, Torimura T, Tanabe N, Aikata H (2020). Randomised, multicentre prospective trial of transarterial chemoembolisation (TACE) plus sorafenib as compared with TACE alone in patients with hepatocellular carcinoma: TACTICS trial. Gut.

[24] Zhao HT, Cai JQ (2021). Chinese expert consensus on neoadjuvant and conversion therapies for hepatocellular carcinoma. World J Gastroenterol.

[25] Shindoh J, Kawamura Y, Kobayashi Y, Kobayashi M, Akuta N, Okubo S (2021). Prognostic impact of surgical intervention after lenvatinib treatment for advanced hepatocellular carcinoma. Ann Surg Oncol.

[26] Ohya Y, Hayashida S, Tsuji A, Kuramoto K, Shibata H, Setoyama H (2020). Conversion hepatectomy for advanced hepatocellular carcinoma after right portal vein transection and lenvatinib therapy. Surg Case Rep.

[27] Sato N, Beppu T, Kinoshita K, Yuki H, Suyama K, Chiyonaga S (2019). Conversion hepatectomy for huge hepatocellular carcinoma with arterioportal shunt after chemoembolization and lenvatinib therapy. Anticancer Res.

[28] Takeda K, Tsurumaru Y, Yamamoto Y, Araki K, Kogure Y, Mori K (2020). Treatment of hepatocellular carcinoma with hepatic vein tumor thrombosis protruding into the inferior vena cava by conversion surgery following chemotherapy with regorafenib: a case report. Clin J Gastroenterol.

[29] Tomonari T, Sato Y, Tanaka H, Tanaka T, Taniguchi T, Sogabe M (2020). Conversion therapy for unresectable hepatocellular carcinoma after lenvatinib: three case reports. Medicine (Baltimore).

